# Belumosudil in diffuse cutaneous systemic sclerosis: a randomized, double-blind, open-label extension, placebo-controlled, phase 2 study

**DOI:** 10.1093/rheumatology/keaf062

**Published:** 2025-03-14

**Authors:** Lorinda Chung, Richard M Silver, Virginia Steen, Daniel E Furst, Flavia V Castelino, Marcin Trojanowski, Robert Spiera, Robyn Domsic, Alicia Rodriguez-Pla, Tamiko R Katsumoto, Helene Goulaouic, Hong Wang, Melanie Espinasse, Souheil El-Chemaly, Rui Wang

**Affiliations:** Department of Medicine and Dermatology, Stanford University School of Medicine, Stanford, CA, USA; Palo Alto VA Health Care System, Palo Alto, CA, USA; Department of Medicine, Medical University of South Carolina (MUSC), Charleston, SC, USA; Department of Medicine, Georgetown University, Washington, DC, USA; Southern California Scleroderma and Rheumatology Centre, Los Angeles, CA, USA; Division of Rheumatology, Massachusetts General Hospital (MGH), Boston, MA, USA; Department of Medicine, Boston University, Boston, MA, USA; Department of Medicine, Hospital of Special Surgery, New York, NY, USA; Division of Rheumatology, Department of Internal Medicine, University of Pittsburgh School of Medicine, Pittsburgh, PA, USA; Division of Rheumatology, Mayo Clinic, Scottsdale, AZ, USA; Department of Medicine and Dermatology, Stanford University School of Medicine, Stanford, CA, USA; Sanofi, Chilly-Mazarin, France; Sanofi, Cambridge, MA, USA; Sanofi, Chilly-Mazarin, France; Sanofi, Cambridge, MA, USA; Sanofi, Cambridge, MA, USA

**Keywords:** belumosudil, diffuse cutaneous systemic sclerosis, CRISS, biomarker, clinical trial

## Abstract

**Objectives:**

To determine the efficacy, safety and pharmacodynamics of belumosudil in patients with diffuse cutaneous systemic sclerosis (dcSSc) treated with background immunosuppressive therapies.

**Methods:**

Eligible patients were randomised 1:1:1 to receive belumosudil 200 mg once daily (QD) or twice daily (BID), or placebo for 28 weeks (double-blind period). After unblinding, the patients who received belumosudil continued the same dose, whereas the patients who received placebo were re-randomised for one of the belumosudil doses for 24 weeks (open-label extension).

**Results:**

Thirty-five and 31 patients were treated in the double-blind and open-label periods, respectively. The study was terminated prematurely, and target enrolment was not met. The primary end point, of CRISS score ≥0.60 at week 24, did not exhibit an efficacy signal in the belumosudil *vs* placebo groups [odds ratio: 1.06 (0.19–5.82; *P* = 0.9472) for the QD, and 0.39 (0.07–2.35; *P* = 0.3078) for the BID group]. Belumosudil was well tolerated and exhibited similar safety profiles in both double-blind and open-label periods. Tissue-based RNA sequencing analysis revealed FOXP3 upregulation and STAT3, IL23A and TGF-β downregulation in patients with CRISS score ≥0.60, which supported the mechanism of action of belumosudil. In blood and tissue samples, trends of decreased fibrosis biomarker levels were seen in the belumosudil-treated group *vs* placebo.

**Conclusion:**

Efficacy signal for belumosudil could not be detected. Signalling pathway modulation analysis supported the mechanism of action of belumosudil. A trend for decreased fibrosis-related biomarkers was observed in the belumosudil-treated group.

**Trial registration:**

ClinicalTrials.gov, https://clinicaltrials.gov, NCT03919799.

Rheumatology key messages

- Given the limited sample size, efficacy signals (CRISS score ≥0.60) were not observed in patients with dcSSc upon belumosudil treatment.
- Signalling pathway modulation analyses supported the mechanism of action of belumosudil.
- Belumosudil was well tolerated in patients with dcSSc.

## Introduction

Systemic sclerosis (SSc; scleroderma) is an orphan, chronic autoimmune disease of unknown aetiology and characterised by organ fibrosis, immune dysregulation and vasculopathy [1, 2]. Diffuse cutaneous SSc (dcSSc) involves proximal skin thickening on the limbs and/or trunk, with variable involvement of the lungs, heart, kidneys, gastrointestinal tract and musculoskeletal system [3–5]. dsSSc has a 10-year mortality rate of 50% [6].

In dcSSc, T cells contribute to disease pathogenesis, evidenced by increased Th17 cells producing interleukin-17 and decreased regulatory T cells [7–9]. Th17 cells and Tregs are associated with upregulation of inflammation and immunosuppressive effect, respectively [8, 9]. Overall, an imbalanced Th17/Treg ratio is reported in patients with SSc [7]. Increased levels of extracellular signalling molecules, such as TGF-β, IL-6 and IL-1 have been reported in tissue and serum samples of patients with SSc, and they are likely involved in the development of a pro-fibrotic state in dcSSc through fibroblast activation, proliferation and collagen production [10].

The current disease-modifying treatment options for SSc, such as immunosuppressive drugs, anti-fibrotic medications and haematopoietic stem cell transplantation (HSCT) are limited and may cause deleterious side effects on vital organs and the immune system. The treatments are often accompanied by poor tolerability and risk of infections and cytopenia [11–13] and pose significant risk of treatment and post-treatment morbidity and mortality [14], creating an unmet need for new and safer therapeutic options for SSc.

Belumosudil is a selective inhibitor of Rho-associated coiled-coil-containing protein kinase-2 (ROCK2) [15]. ROCK2 inhibition downregulates STAT3 phosphorylation and Th17 cell-specific transcription factors. ROCK2 inhibition by belumosudil maintains immune homeostasis by shifting the Th17/Treg balance toward Tregs via a STAT5-dependent mechanism [16]. ROCK2 is also a key factor in the development of pulmonary fibrosis, where it functions downstream of several major pro-fibrotic mediators, including TGF-β, connective tissue growth factor and lysophosphatidic acid [17]. In a preclinical study, belumosudil reduced lung fibrosis in animal models of chronic graft *vs* host disease (cGVHD) and inhibited the production of IL-21, IL-17 and IFN-γ in *ex-vivo* peripheral blood mononuclear cells (PBMCs) [18]. In a Phase 2 randomised clinical trial of patients with cGVHD requiring third- or later-line therapy (*n* = 132), 200 mg belumosudil once daily (QD) or twice daily (BID) doses were well tolerated and demonstrated efficacy in the patients, including patients with cGVHD refractory to corticosteroids, ruxolitinib and ibrutinib. Subsequently, in July 2021, belumosudil was approved by the Food and Drug Administration (FDA) for use in adult/paediatric patients with cGVHD after failure of two lines of systemic therapy [19].

Thus, based on the substantial unmet need for therapies with improved tolerability and effectiveness, we initiated this Phase 2 study to determine the efficacy, safety and pharmacodynamics (PD) of orally administered belumosudil in patients with dcSSc.

## Patients and methods

### Study design and treatment

This randomised, placebo-controlled Phase 2 study involved patients with dcSSc and was conducted at 26 centres across the USA. A double-blind 28-week period was followed by an open-label extension of 24 weeks. The duration of the study was ∼14 months with 4-week screening, 52-week dosing and 4-week follow-up periods (Supplementary Fig. S1, available at *Rheumatology* online). Eligible patients were randomised (1:1:1) to either receive oral belumosudil (200 mg QD or 200 mg BID) or matched placebo in the double-blind period. In the open-label period, the participants in the belumosudil groups continued with the same dose and schedule, whereas the participants in the placebo group were re-randomised (1:1) to receive one of the two belumosudil doses (200 mg QD/200 mg BID).

### Patients

Adult patients who met the 2013 American College of Rheumatology (ACR) and European League Against Rheumatism (EULAR) criteria for dcSSc [20], with disease duration (defined as the interval from the first non-Raynaud disease manifestation) of ≤5 years, modified Rodnan skin score (mRSS) ≥15 but ≤35 and active disease within 6 months prior to screening were included in the study. Active disease was defined as increase in mRSS by ≥3 units; increase in mRSS by ≥2 units with involvement of one new body area; involvement of two new body areas; or symptoms indicative of skin activity. Protocol-specified ongoing immunosuppressive therapies (IST) were allowed if the patient was on a stable dose for the past 6 months.

The key exclusion criteria were forced vital capacity (FVC) ≤50% predicted and scleroderma renal crisis within 4 months before enrolling. Detailed inclusion and exclusion criteria are listed in Table S1.

### Study endpoints

The primary end point was the number of patients with combined response index in systemic sclerosis (CRISS) score ≥0.60 at week 24 and continuous CRISS score at week 24. The ACR CRISS is a composite end point that captures cardio–pulmonary–renal involvement and change in mRSS, Scleroderma Health Assessment Questionnaire Disability-Index (SHAQ-DI), patient global assessment (PaGA), physician global assessment scale (PGA) and FVC% predicted value [21].

Secondary endpoints were continuous CRISS score at week 52; mean value, change from baseline, and percentage improvement at week 24 and at week 52 in mRSS, FVC% predicted, PGA, PaGA and SHAQ-DI; and mean value, change from baseline and percentage improvement at week 52 in pulmonary function test (PFT) in patients with interstitial lung disease (ILD) at screening. Lung fibrosis was assessed via high-resolution computerised tomography (HRCT) at week 24 and at week 52 in patients with ILD at screening, and safety was assessed based on the percentage of patients who experienced treatment-emergent adverse events (TEAEs) and treatment-emergent serious adverse events [SAEs; common terminology criteria for adverse events (AEs) version 5.0 (CTCAE v5.0)]. The exploratory endpoints included changes in biomarker levels of endothelial cell dysfunction, fibrosis and immune system function, and cytokine alterations in patients who received belumosudil compared with those of the placebo group, and changes in dermal histology and gene expression. The study endpoints and objectives are described in detail in Supplementary Table S2, available at *Rheumatology* online.

### Statistical analysis

#### Sample size determination

The study was planned to enrol 60 participants. Sample size and power were not driven by hypothesis testing because this was an exploratory study.

#### Efficacy analyses

Efficacy was analysed for the modified intent-to-treat (mITT) population, which included all patients who received at least one dose of the study drug. CRISS score ≥0.20 was the minimal detectable difference and CRISS score ≥0.60 was the minimal clinically important difference [22]. The number and percentage of patients with CRISS score ≥0.60 were summarised by treatment group. CRISS ≥0.60 responses were analysed using logistic regression analysis with treatment in the model. The proportions and 95% CI [Clopper-Pearson (exact) method] were calculated. Continuous CRISS score was assessed using the mixed-effects model of repeated measures (MMRM) model and *t* test comparison at all visits, including unscheduled visits through week 52. In case any component score was missing, the linearisation and last observation carried forward (LOCF) method was used for imputing CRISS score. For the secondary endpoints with continuous variables (mRSS, PFTs, PGA/PaGA, SHAQ-DI), the changes from baseline value and percentage improvement were also assessed using the MMRM model. In addition to the MMRM model, treatment comparisons for continuous efficacy variables were assessed using an analysis of covariance (ANCOVA) model, which included treatment groups and baseline values.

#### Safety analyses

All safety evaluations were performed for the safety population, which was equivalent to the mITT population in this study. AEs were summarised using descriptive statistics and were coded using the MedDRA dictionary (v24.0). TEAEs were defined as occurrence or worsening in severity of any AE after the first administration of study medication. All AEs, including SAEs, were graded using the 5-point CTCAE v5.0 scale (mild, moderate, severe, life-threatening or death). Causality due to study treatment was classified as definitely related; probably related; possibly related; unlikely related; or not related.

The number (N) and percentage (%) of patients who experienced at least one TEAE were summarised by dose group (belumosudil 200 mg QD/belumosudil 200 mg BID/placebo).

#### Biomarker analyses

*RNA-sequencing (RNA-seq) and data processing.* Total RNA was isolated from 4-mm skin biopsies. RNeasy fibrous tissue kits (Qiagen, Germantown, Maryland, USA) were used for RNA extraction, and rRNA reduction (ribodepletion) was performed before RNA-seq at 100-bp read length to reach 100M PE reads (200M total reads) on a NovaSeq 6000 system (Illumina, San Diego, California, USA). Median sequencing depth was 114.3 million, with the median mapping rate to the genome of 99.5%. Of the total number of mapped reads, median 40.9% reads were mapped to exonic sequences. Quality checks on raw sequence data for each sample were performed using FastQC. Raw reads were mapped to the reference hg38 (GRCh38.p13 with alternative loci and HLA decoy sequences) genome, using the DRAGEN pipeline v3.10 (Illumina, Sandiego, California, USA). The alignment metrics of the mapped reads were estimated using Picard tools. Aligned reads were imported to a commercial data analysis platform Avadis next-generation gene sequencing. After quality inspection, the aligned reads were filtered based on read quality metrics, and reads with a base quality score <30, alignment score <95 and mapping quality <40 were removed. The remaining reads were filtered based on their read statistics, and missing mates, translocated, unaligned, and flipped reads were removed. The reads list was filtered to remove duplicates. For each sample, raw sequence FASTQ, binary alignment map and transcript/gene count matrix files [NumReads, fragments per kilobase per million mapped reads (FPKM) and transcript per million were generated using feature Counts and GENCODE V4.0].

*Differential expression and pathway analysis.* Raw read counts were used to analyse differential gene expression using the DESeq2 module from GenePattern. Differentially expressed genes (DEGs) with false discovery rate (FDR) ≤5% were considered significant. Normalised counts generated using the DESeq2 module were used as input for gene set enrichment analysis (GSEA) module from GenePattern. Genes were annotated to functional terms using: Profiler run *vs* Gene Ontology and Biological Pathways databases. Samples were assigned to three molecular subsets (inflammatory, fibroproliferative and normal-like), using subset-specific gene signatures and the FPKM matrix.

Molecular subsets at baseline and week 24 were illustrated by using the Sankey plot.

Boxplots with data points were used to visualise the distribution of PD biomarkers at each time point (baseline, week 24 and week 52) and to reveal the change in trend after treatment.

### Ethics approval

The study was approved by all local institutional review boards and performed according to the Declaration of Helsinki and International Conference on Harmonization Good Clinical Practice guidelines. All participants provided written informed consent.

## Results

### Patient disposition and baseline characteristics

**Figure 1.**
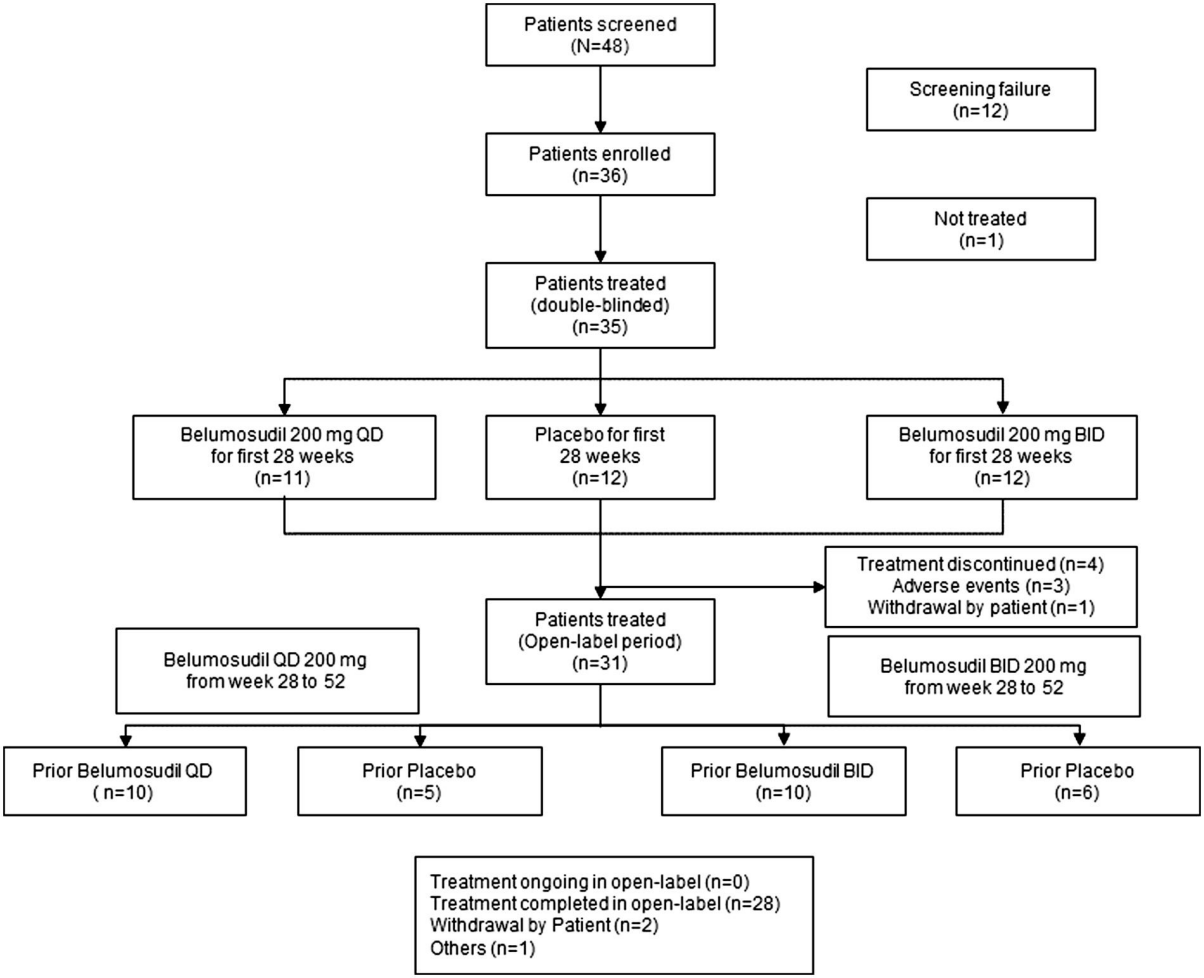
CONSORT diagram for patient disposition in the treated population. BID: twice daily; CONSORT: consolidated standards of reporting trials; N/n: number of patients; QD: once daily

From 9 July 2019 to 24 December 2022, 35 and 31 patients were treated in the double-blind and open-label periods, respectively (Fig. 1). Demographic and baseline characteristics are summarised in Table 1. The enrolled patients had a median age of 49.0 years and were predominately females (26 [74.3%]). Seventeen (49.6%) patients had a history of ILD. The median scores at baseline for mRSS, SHAQ-DI, PGA and PaGA were 24.0 (15.0–35.0), 1.4 (0.0–2.8), 61.0 (10.0–86.0) and 44.0 (20.0–79.0), respectively. Most patients (33 [94.3%]) received ≥1 concomitant immunosuppressant, of which mycophenolate was the most common (28/35; 80%) followed by methotrexate (6/35; 17.1%).

**Table 1.** Patients’ baseline demographics and disease characteristics

	Belumosudil 200 mg QD	Belumosudil 200 mg BID	Belumosudil 200 mg (QD + BID)	Placebo	All
	(*n*=11)	(*n*=12)	(*n*=23)	(*n*=12)	(N=35)
Age (years)					
Median (range)	55.0 (29–72)	49.0 (27–78)	50.0 (27–78)	45.0 (30–67)	49.0 (27–78)
Gender, *n* (%)					
Male	4 (36.4)	3 (25.0)	7 (30.4)	2 (16.7)	9 (25.7%)
Female	7 (63.6)	9 (75.0)	16 (69.6)	10 (83.3)	26 (74.3%)
Race, *n* (%)					
Asian	2 (18.2)	0	2 (8.7)	0	2 (5.7%)
White	8 (72.7)	8 (66.7)	16 (69.6)	11 (91.7)	27 (77.1%)
Black or African American	1 (9.1)	3 (25.0)	4 (17.4)	0	4 (11.4%)
Unreported or unknown	0	1 (8.3)	1 (4.3)	1 (8.3)	2 (5.7%)
History of interstitial lung disease					
Yes	4 (36.4)	7 (58.3)	11 (47.8)	6 (50.0)	17 (48.6%)
No	7 (63.6)	5 (41.7)	12 (52.2)	6 (50.0)	18 (51.4%)
mRSS					
Median (range)	29 (15–35)	24 (16–34)	26 (15–35)	22 (16–30)	24 (15–35)
SHAQ-DI					
Median (range)	1.6 (0.0–2.8)	1.4 (0.1–2.3)	1.5 (0.0–2.8)	1.2 (0.3–2.4)	1.4 (0.0–2.8)
Patient global assessment					
Median (range)	56.0 (10–86)	64.0 (24–78)	62.0 (10–86)	59.5 (38–84)	61.0 (10–86)
Physician global assessment					
Median (range)	38.0 (20–73)	59.5 (36–74)	51.0 (20–74)	38.0 (25–79)	44.0 (20–79)
Patients on at least one concomitant immunosuppressant medication					
*n* (%)	11 (100.0)	11 (91.7)	22 (95.7)	11 (91.7)	33 (94.3)
Mycophenolate	10 (72.7)	8 (66.7)	18 (69.6)	10 (75.0)	28 (80.0)
Glucocorticosteroids	4 (36.3)	5 (41.6)	9 (39.1)	4 (33.3)	13 (37.1)
Methotrexate	1 (9.1)	4 (33.3)	5 (21.7)	1 (8.3)	6 (17.1)
D-Penicillamine	0	1 (8.3)	1 (4.3)	1 (8.3)	2 (5.8)
Hydroxychloroquine	1 (9.1)	1 (8.3)	2 (8.7)	1 (8.3)	3 (8.6)
Tocilizumab	1 (9.1)	0	1 (4.3)	0	1 (2.9)
Rituximab	0	1 (8.3)	1 (4.3)	0	1 (2.9)
IVIG	0	0	0	1 (8.3)	1 (2.9)

BID: twice daily; IVIG: intravenous immunoglobulin; mRSS: modified Rodnan skin score; n/N: number of patients; QD: once daily; SHAQ-DI: scleroderma health assessment questionnaire disability-index.

### Efficacy

The primary end point analysis (CRISS score ≥0.60 at week 24) did not show an efficacy signal in the belumosudil-treated *vs* placebo-treated groups. Odds ratio for the QD and BID groups was 1.06 (0.19–5.82; *P* = 0.9472) and 0.39 (0.07–2.35; *P* = 0.3078), respectively (Table 2). The number of patients with CRISS score ≥0.60 at week 24 was similar in the belumosudil QD and placebo groups (6 [54.5%] and 7 [58.3%], respectively), but it was numerically lower in the belumosudil BID group (3 [25.0%]) as per the LOCF analysis. Statistically significant difference was not observed in the continuous CRISS score at week 24 between the groups. The least square mean (LSM) difference (95% CI) between the placebo, belumosudil QD and belumosudil BID groups was −0.04 (−6.51–6.42; *P* = 0.9889) and −4.18 (−10.59–2.23; *P* = 0.1916), respectively (Table 2). The lowest mean CRISS scores at all timepoints (excluding week 8) were observed in patients in the belumosudil BID group, whereas the patients in the belumosudil QD group had the highest mean CRISS scores at week 24 as per the LOCF analysis.

**Table 2.** Analysis of CRISS score and continuous CRISS score for the first 24 weeks

	Belumosudil 200 mg QD	Belumosudil 200 mg BID	Belumosudil 200 mg (QD + BID)	Placebo
	(*n*=11)	(*n*=12)	(N=23)	(*n*=12)
CRISS Score (mITT population), *n* (%)				
≥0.60	3 (27.3%)	2 (16.7%)	5 (21.7%)	6 (50.0%)
95% CI of ≥0.60^a^	(6.0%, 61.0%)	(2.1%, 48.4%)	(7.5%, 43.7%)	(21.1%, 78.9%)
CRISS Score (LOCF), *n* (%)				
≥0.60	6 (54.5%)	3 (25.0%)	9 (39.1%)	7 (58.3%)
95% CI of ≥0.60^a^	(23.4%, 83.3%)	(5.5%, 57.2%)	(19.7%, 61.5%)	(27.7%, 84.8%)
CRISS Score≥0.60 (LOCF) comparison using logistic regression				
Odds ratio (95% CI) vs placebo	1.06 (0.19, 5.82)	0.39 (0.07, 2.35)	0.66 (0.15, 2.85)	—
*P*-value vs. placebo	0.9472	0.3078	0.5810	
Odds ratio (95% CI) vs. belumosudil (QD)	—	0.37 (0.06, 2.40)	—	—
*P*-value vs. KQD		0.2989		
CRISS score at Week 24 evaluated using MMRM model^b^				
LSM difference (95% CI) vs placebo	−0.04 (−6.51, 6.42)	−4.18 (−10.59, 2.23)	—	—
*P*-value vs placebo	0.9889	0.1916	—	—

a Confidence intervals are constructed using the simple asymptotic method, without continuity correction (i.e. normal approximation to the binomial distribution). CRISS score >0.60 is the minimally important difference and has been used for this analysis. CRISS score was imputed using LOCF in case of missing data.

b MMRM analysis was performed using rank-transformed data, with CRISS score as a dependent variable and treatment, visit, and visit × treatment interaction as fixed effects. Visit was a repeated factor, and analyses were done through week 24.

BID: twice daily; CI: confidence interval; CRISS: Combined Response Index in Diffuse Cutaneous Systemic Sclerosis; LOCF: lack of efficacy; LSM: least square mean; mITT: modified intent-to-treat; n/N: number of patients; QD: once daily; *vs*: *vs.*

We did not observe a significant difference in any CRISS score component from baseline to week 24 between patients treated with belumosudil and the placebo group. A trend toward improved response was observed for PGA score between belumosudil QD and the placebo groups from baseline to week 24 (LSM difference [95% CI] 14.6 [−2.5–31.8]; *P* = 0.0916). However, improvement was not observed in the belumosudil BID group *vs* the placebo group (LSM difference [95% CI] −8.6 [−25.7–8.6]; *P* = 0.3146). For mRSS, decrease in LSM from baseline to week 24 was similar between the belumosudil QD and placebo groups but was lower in the belumosudil BID group, indicating significant improvement in the placebo group *vs* the belumosudil BID group (*P* = 0.0308) (Supplementary Table S3; and Supplementary Fig. S2, available at *Rheumatology* online).

PFT parameters, including FVC in the overall population and in patients with underlying ILD, did not exhibit a clear trend due to the small sample size ILD population. In patients with ILD, change in FVC and diffusing capacity of the lungs for carbon monoxide (DLco) were similar across the treatment groups from baseline to week 24. The improvement in total lung capacity (TLC) was numerically better in the placebo group (11.0 [3–17]) *vs* the belumosudil groups (QD; −14.0 [−14– −6] and BID; −2.0 [−8–2]); however, the difference was not significant (Supplementary Table S4, available at *Rheumatology* online).

### Safety

A similar safety profile was observed during the double-blind and open-label periods, and deaths or life-threatening TEAEs were not reported during the study. Overall, 33 (94.3%) patients experienced AEs and TEAEs during the double-blind period, with 11 patients each in the QD, BID and placebo arms. Five (14.3%) patients experienced serious TEAEs in the QD (2 [18.2%]) and placebo (3 [25.0%]) arms, whereas Grade ≥3 TEAEs were experienced by 3 (8.6%) patients, i.e. 1 (8.3%) and 2 (16.7%) in the BID and placebo arms, respectively. Eighteen (51.4%) patients experienced treatment-related adverse events (TRAEs); 7 (63.6%), 6 (50.0%) and 5 (41.7%) in the QD, BID and placebo groups, respectively. In the open-label period, 28/31 (90.3%) patients experienced AEs and TEAEs. One (6.3%) patient experienced a serious TEAE in the BID group, and 3 (9.7%) patients experienced Grade ≥3 TEAEs: 1 (6.7%) and 2 (12.5%) in the QD and BID groups, respectively. Five (31.3%) patients experienced TRAEs in the BID group (Supplementary Table S5, available at *Rheumatology* online).

The most common TEAEs (that occurred in ≥10% patients) in the double-blind period were nausea, headache and diarrhoea, which were observed in 9 (25.7%), 9 (25.7%) and 7 (20.0%) patients in the QD, BID and placebo groups, respectively. The QD group reported more TEAEs related to diarrhoea (4 [36.4%]) than the other treatment groups, and the placebo group reported the most TEAEs related to headache (7 [58.3%]) (Table 3). Grade ≥3 TEAE were COVID-19 (1 [8.3%]) in the BID group and anaemia, scleroderma renal crisis and pulmonary oedema in the placebo group (reported by 1 patient each; 8.3%) (Supplementary Table S6, available at *Rheumatology* online).

**Table 3.** Summary of treatment-emergent adverse events (TEAEs) observed in ≥10% of patients in the safety population

N (%)	Double-blind period^a^			Open-label period^b^	
	Belumosudil 200 mg QD	Belumosudil 200 mg BID	Placebo	Belumosudil 200 mg QD	Belumosudil 200 mg BID
	(*n*=11)	(*n*=12)	(*n*=12)	(*n*=15)	(*n*=16)
All TEAEs	11 (100.0)	11 (91.7)	11 (91.7)	14 (93.3)	14 (87.5)
Gastrointestinal disorders	8 (72.7)	4 (33.3)	9 (75.0)	6 (40.0)	7 (43.8)
Nausea	4 (36.4)	0	5 (41.7)	2 (13.3)	0
Dysphagia	0	0	0	2 (13.3)	0
Diarrhoea	4 (36.4)	0	2 (16.7)	3 (20.0)	2 (12.5)
Gastroesophageal reflux disease	2 (18.2)	2 (16.7)	0	0	2 (12.5)
Vomiting	2 (18.2)	0	0	0	2 (12.5)
Constipation	0	0	3 (25.0)	0	0
Skin and subcutaneous tissue disorder	5 (45.5)	3 (25.0)	6 (50.0)	6 (40.0)	4 (25.0)
Pruritus	3 (27.3)	0	3 (25.0)	0	0
Rash	0	2 (16.7)	0	0	0
Infections and infestations	5 (45.5)	4 (33.3)	4 (33.3)	5 (33.3)	5 (31.3)
COVID-19	2 (18.2)	2 (16.7)	0	3 (20.0)	3 (18.8)
Upper respiratory tract infection	0	2 (16.7)	0	0	0
Nervous system disorders	4 (36.4)	2 (16.7)	7 (58.3)	4 (26.7)	2 (12.5)
Headache	2 (18.2)	0	7 (58.3)	0	0
Dizziness	0	0	2 (16.7)	0	2 (12.5)
Hypoesthesia	2 (18.2)	0	0	0	0
General disorders and administration site conditions	3 (27.3)	3 (25.0)	3 (25.0)	2 (13.3)	6 (37.6)
Fatigue	2 (18.2)	0	2 (16.7)	0	2 (12.5)
Musculoskeletal and connective tissue disorders	2 (18.2)	4 (33.3)	2 (16.7)	2 (13.3)	3 (18.8)
Pain in extremity	0	2 (16.7)	0	0	0
Respiratory, thoracic, and mediastinal disorders	2 (18.2)	3 (25.0)	3 (25.0)	0	5 (31.3)
Dyspnoea exertional	0	2 (16.7)	0	0	2 (12.5)
Cough	0	0	0	0	2 (12.5)
Epistaxis	0	0	2 (16.7)	0	0
Vascular disorders	0	0	4 (33.3)	0	0
Raynaud’s phenomenon	0	0	3 (25.0)	0	0
Hypertension	0	0	0	0	0
Blood and lymphatic system disorders	0	0	3 (25.0)	0	2 (12.5)
Anaemia	0	0	2 (16.7)	0	0
Psychiatric disorders	0	0	2 (16.7)	0	3 (18.8)
Anxiety	0	0	2 (16.7)	0	0
Depression	0	0	0	0	2 (12.5)
Insomnia	0	0	0	0	2 (12.5)

a For double-blinded period, belumosudil 200 mg QD or belumosudil 200 mg BID or placebo were administered for the first 28 weeks.

b For open-label period belumosudil 200 mg QD or belumosudil 200 mg BID were administered from week 28 to week 52.

BID: twice daily; COVID: coronavirus disease; *n*: number of patients; QD: once daily.

In the open-label period, the most common TEAEs (that occurred in ≥10% patients) in the QD group were diarrhoea and COVID-19, each experienced by 3 (20%) patients, followed by nausea and dysphagia reported in 2 (13.3%) patients each. The most common TEAE in the BID group was COVID-19, experienced by 3 (18.8%) patients (Table 3). Three patients reported at least one Grade ≥3 TEAE (hypertension, bradycardia, congestive cardiac failure, ischaemic cardiomyopathy and exertional dyspnoea) that was not related to study treatment (Supplementary Table S6, available at *Rheumatology* online).

### Biomarker analyses

**Figure 2.**
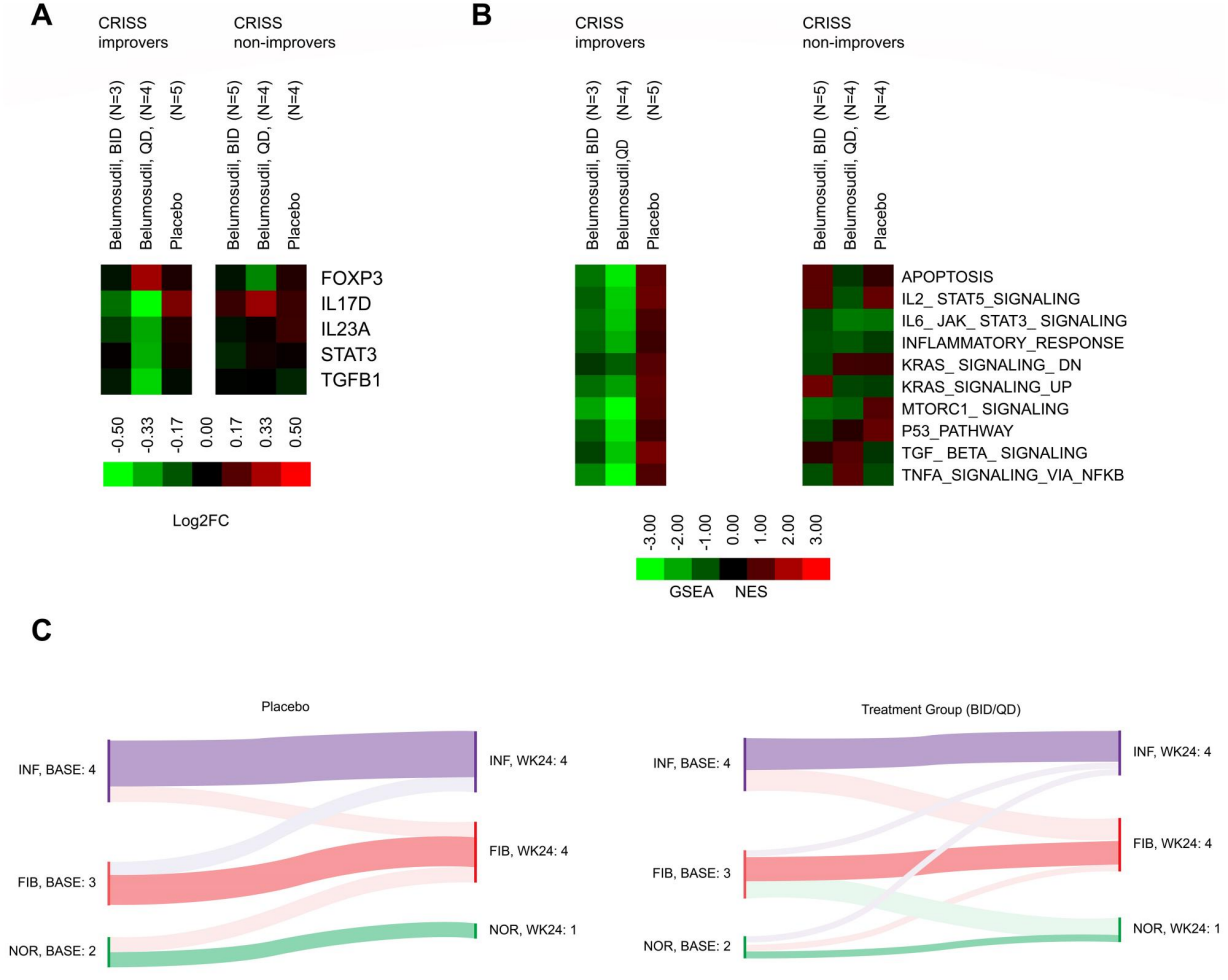
Signalling pathway modulation observed in tissue samples analysed using RNA sequencing. (**A**) Heatmap^a,b^ representing the summary analysis of gene expression changes across treatment arms. (**B**) Heatmap^a,c^ representing the summary of pathway changes across treatment arms. (**C**) Phenotype assessment in paired biopsies collected from placebo and belumosudil-treated groups (BID/QD), using RNA sequencing. ^a^Heatmaps colour coding: Green = significant decrease at Week 24 versus the baseline; red = significant increase at Week 24 versus the baseline; black = NSC. ^b^Column = treatment arm (all participants or stratified by clinical response), row = individual gene. Cell log2 fold change representing the significance of enrichment at Week 24 relative to baseline. ^c^Column = treatment arm stratified by clinical response. Row = pathway (gene set) from Hallmarks gene set database. Cell = NES of a particular pathway representing the significance of enrichment at Week 24 versus the baseline. BASE: baseline; BID: twice daily; CRISS: Combined Response Index in Diffuse Cutaneous Systemic Sclerosis; FIB: fibroproliferative; GSEA: gene set enrichment analysis; IL: interleukin; KRAS: Kirsten rat sarcoma virus; MoA: mechanism of action; MTORC1: mammalian target of rapamycin complex 1; NES: normalised enrichment score; NFKB: nuclear factor kappa B; NOR: normal-like; NSC: no significant change; QD: once daily; TGF: transforming growth factor; TNFA: tumour necrosis factor alpha; WK: week

RNA-seq data revealed FOXP3 (a marker for Tregs) upregulation and STAT3/interleukin 23A/TGF-β downregulation, particularly in the improvers (CRISS ≥0.60) who were treated with belumosudil 200 mg QD; these changes were not reported in the placebo group (Fig. 2A). Moreover, downregulation of pathway signature genes was observed in the improvers, supporting the mechanism of action of belumosudil (as a ROCK2 inhibitor) and suggesting the potential of response signature identification in the improvers (Fig. 2B).

RNA-seq analysis revealed three phenotypes of the paired biopsies, namely inflammatory, fibroproliferative and normal-like; most patients retained the same phenotype after treatment with either belumosudil (BID/QD) or placebo (Fig. 2C).

**Figure 3.**
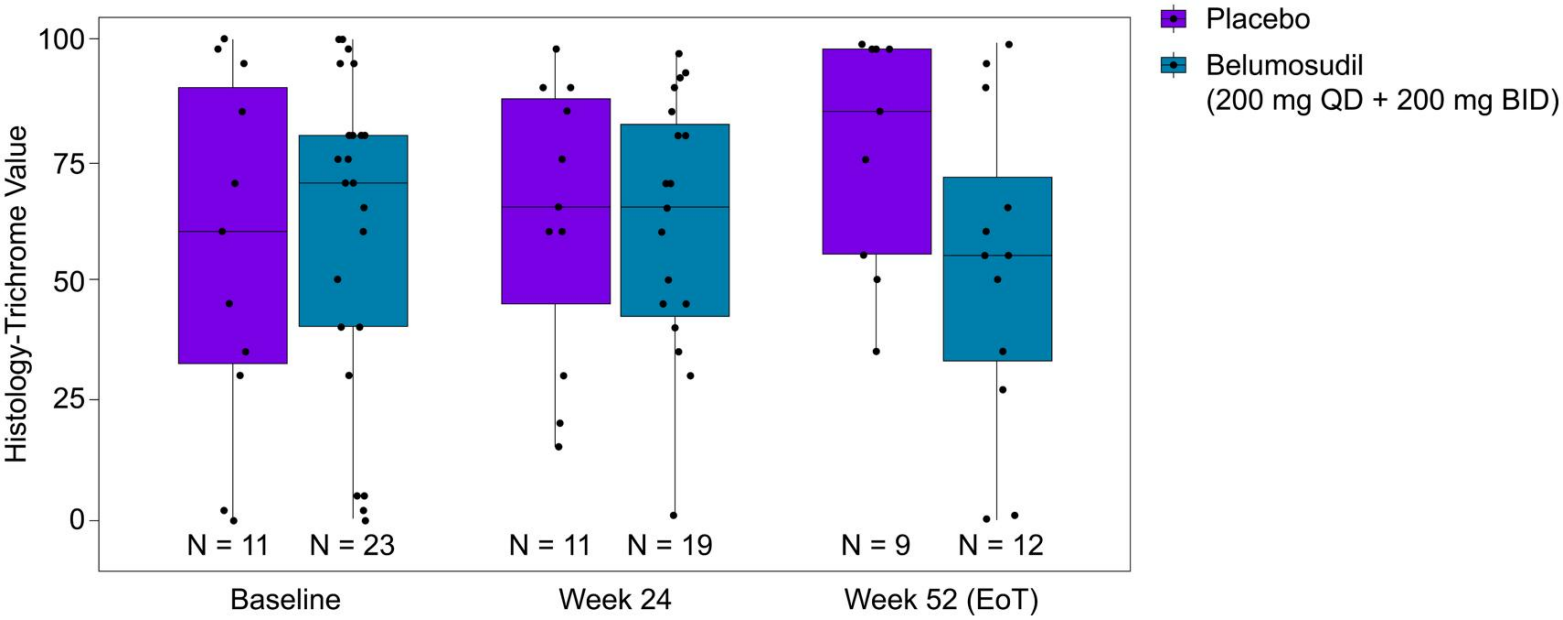
Treatment-related impact on tissue fibrosis in patients treated with belumosudil *vs* placebo till week 52. The analysis was done using Masson’s trichome staining. Box centre and upper/lower lines indicate the median and upper/lower quartile, respectively. Vertical lines above and below the box indicate 1.5 times the interquartile range. EOT: end of treatment; ID: twice daily; N: number of patients; QD: once daily

Furthermore, our histologic analysis revealed decreased fibrosis at week 24 and week 52 (end of treatment) in belumosudil-treated patients (both QD and BID) compared with that in the placebo group (Fig. 3).

Enhanced liver fibrosis (ELF) scores for all three biomarkers (i.e. HA, PNIIIP and TIMP-1) exhibited a decreasing trend in the treated groups compared with that in the placebo group at baseline, week 4, week 8 and week 24 (Supplementary Fig. S3, available at *Rheumatology* online).

## Discussion

This placebo-controlled, double-blinded, randomised Phase 2 study assessed the efficacy of orally administered belumosudil in patients with dcSSc. Efficacy was evaluated based on ACR CRISS ≥0.60 at week 24 as the primary end point, and most patients were treated with background ISTs. The study was terminated prematurely due to slow enrolment and not to any safety concerns; therefore, target enrolment of 60 patients was not achieved. As such, an important limitation of these efficacy findings is the small and underpowered sample size.

This study, like the RESOLVE-1 randomised Phase 3 study [23] that investigated the efficacy and safety of lenabasum (a cannabinoid type 2 receptor agonist) in patients with dcSSc, used the ACR CRISS score as the primary efficacy outcome and allowed patients to receive background ISTs. In both studies, the study treatment and placebo groups could not be differentiated based on ACR CRISS score. This may be because of the ceiling effect that is observed with background ISTs, particularly mycophenolate mofetil, that could have impacted the CRISS score in the context of a clinical trial. In two other SSc trials – the abatacept study [1], where the ACR CRISS score was used as a secondary end point, and the tocilizumab study [24] where it was an exploratory end point – the ACR CRISS score discriminated the active drug from placebo. However, the patients in neither of these trials were allowed to be on concomitant background ISTs.

In the present study, biomarker analysis based on tissue-based RNA-seq revealed an upregulation of FOXP3 and downregulation of STAT3, IL23A and TGF-β in patients who achieved a CRISS score ≥0.60 (improver population). These findings are in line with the observations from a Phase 2 study on patients with cGVHD [25], wherein early increase in the percentage of CD4^+^ Tregs among PBMCs was observed upon belumosudil treatment, accompanied by a simultaneous decrease in Th17 cell number. Other studies have also reported similar observations that ROCK2 inhibition by belumosudil impacted immune homeostasis of Th17/Tregs, shifting the immune milieu toward a larger proportion of Tregs [18]. A clear pattern of pathway signature downregulation was also observed in the improver group, supporting the mechanism of action of belumosudil in this patient population. The sclerotic pathology of cGVHD and dcSSc differ significantly, particularly in capillary changes. In cGVHD, there is early focal capillary proliferation similar to wound healing, whereas dSSc is marked by the loss of dermal capillaries and VE-cadherin. These differences could potentially explain the differential effects of belumosudil in cGVHD and dcSSc [26, 27].

A limited number of serum biomarkers are available for the detection of fibrosis in SSc. Chen C and colleagues [28] used the ELF score as a surrogate outcome measure of fibrosis in 85 patients with SSc. They revealed that serum PIIINP and TIMP-1 levels and ELF score were significantly higher in patients with SSc than in healthy controls. In our study, the ELF score was used as a biomarker to detect potential fibrosis, and a decreasing trend was observed in the treated group *vs* the placebo group. Similarly, histological analysis revealed a decrease in tissue fibrosis following belumosudil treatment. However, the sample size is a limitation, which may account for the lack of significant differences observed in peripheral biomarkers.

Belumosudil showed an acceptable safety profile in patients with dcSSc, which was similar during the open-label and the double-blind periods. Belumosudil appeared to be safe and well tolerated at both doses (QD and BID) *vs* the placebo group. Additionally, there were no deaths or life-threatening events reported during this study. These findings are consistent with previously published studies involving patients with cGVHD [19, 25].

The limitations of this study include the following. Firstly, the patients were allowed to continue background ISTs (such as methotrexate and mycophenolate mofetil), like the RESOLVE-1 dcSSc study [23], possibly causing a ceiling effect, impacting the ACR CRISS score. Hence, future studies should investigate this aspect, and the impact of background ISTs should be considered while designing similar studies on patients with scleroderma. Secondly, the use of the LOCF method for imputing the missing data could have led to biased treatment effects, resulting in biased estimators. Thirdly, the small sample size of this study (31–35 patients enrolled out of 60 planned) limits the interpretation of the biomarker and efficacy analyses and prevents definitive conclusions from being drawn. Furthermore, the broad definition of active disease led to the inclusion of patients with varying severity levels, potentially resulting in a heterogeneous population and difficulty detecting a treatment effect.

Therefore, this prematurely terminated Phase 2 trial could not identify any trend toward belumosudil efficacy in the primary outcome variable and its components, but it was found to have acceptable safety and tolerability. Biomarker analysis supported the mechanism of action of belumosudil and revealed trends of decrease in the levels of fibrosis-related biomarkers and Th17-mediated inflammation associated with ROCK2 inhibition.

## Supplementary material

Supplementary material is available at *Rheumatology* online.

## Supplementary Material

keaf062_Supplementary_Data

## Data Availability

Qualified researchers may request access to patient-level data and related documents (including, e.g. the clinical study report, study protocol with any amendments, blank case report form, statistical analysis plan and dataset specifications). Patient-level data will be anonymized, and study documents will be redacted to protect the privacy of trial participants. Further details on Sanofi's data sharing criteria, eligible studies and process for requesting access can be found at https://vivli.org/.
