# Supplementary material for: Belumosudil in diffuse cutaneous systemic sclerosis: a randomized, double-blind, open-label extension, placebo-controlled, phase 2 study
Source: Rheumatology (Oxford). 2025 Mar 14;64(7):4299–308. doi: 10.1093/rheumatology/keaf062 (PMC12212915; doi:10.1093/rheumatology/keaf062)
Supplement: keaf062_Supplementary_Data [file keaf062_supplementary_data.zip › keaf062_Supplementary_Data/rhe-24-0882-File002.docx]

**Supplemental section**

**Supplementary Table S1:** List of inclusion and exclusion criteria

| **Inclusion criteria** | **Exclusion criteria** |
| --- | --- |
| Male and female subjects ≥ 18 years old with the diagnosis of dcSSc according to the 2013 ACR and EULAR criteria | Patients corrected QT interval using Fredericia’s formula (QTcF) >450 milliseconds |
| Disease duration (defined as interval from first non-Raynaud disease manifestation) of ≤5 years | Ongoing use or current use of concomitant medication known to have the potential for QTc prolongation |
| Had mRSS of ≥15 but ≤35 | Female subject who is pregnant or breastfeeding |
| Active disease defined as any of the following within the 6 months prior to screening:   1. Increase in mRSS by ≥3 units 2. Increase in mRSS by ≥2 units with involvement of 1 new body area 3. Involvement of 2 new body areas 4. Symptoms indicative of skin activity such as severe cutaneous itching or burning | Participated in another study with an investigational drug within 28 days of study entry (for studies involving biologics, within 3 half-lives of the biologic) |
| Patients receiving concomitant immunosuppression must be on a stable dose for at least 3 months prior to screening | History or other evidence of severe illness or any other conditions that would make the subject, in the opinion of the Investigator, unsuitable for the study |
| Adequate organ and bone marrow functions evaluated during the 28 days prior to enrolment as follows:   1. Absolute neutrophil count ≥1.5 × 10^9^/L 2. Platelet count ≥100 × 10^9^/L 3. Total bilirubin ≤1.0 × ULN 4. Alanine aminotransferase, aspartate aminotransferase, and serum creatinine ≤1.5 × ULN | Chronic heart failure with New York Heart Association Classes II, III, and IV |
| Female patients of childbearing potential have a negative pregnancy test at screening. Females of childbearing potential are defined as sexually mature women without prior hysterectomy or who have had any evidence of menses in the past 12 months. However, women who have been amenorrhoeic for 12 or more months are still considered to be of childbearing potential if the amenorrhea is possibly due to prior chemotherapy, anti-oestrogens, or ovarian suppression.   1. Women of childbearing potential (i.e., menstruating women) must have a negative urine pregnancy test (positive urine tests are to be confirmed by serum test) documented within the 24-hour period prior to the first dose of study drug. 2. Sexually active women of childbearing potential enrolled in the study must agree to use 2 forms of accepted methods of contraception during the course of the study and for 3 months after their last dose of study drug. Effective birth control includes (i) IUD plus 1 barrier method; (ii) on stable doses of hormonal contraception for at least 3 months (e.g., oral, injectable, implant, transdermal) plus 1 barrier method; or (iii) two barrier methods. Effective barrier methods are male or female condoms, diaphragms, and spermicides (creams or gels that contain a chemical to kill sperm), or a vasectomized partner | Acute or chronic liver disease (e.g., cirrhosis) |
| For male patients who are sexually active and who are partners of premenopausal women: agreement to use 2 forms of contraception as in criteria above during the treatment period and for at least 3 months after the last dose of study drug. | Positive human immunodeficiency virus (HIV) test |
| Male subjects must not donate sperm for 3 months after last dose of study drug | Active hepatitis C virus (HCV), hepatitis B virus (HBV), or positive whole blood tuberculin test |
| Able to provide written informed consent prior to the performance of any study-specific procedures. | Diagnosed with any malignancy within 3 years of enrolment, with the exception of basal cell or completely resected squamous cell carcinoma of the skin, resected in situ cervical malignancy, resected breast ductal carcinoma in situ, or low-risk prostate cancer after curative resection |
|  | Has had previous exposure to belumosudil or known allergy/sensitivity to belumosudil or any other ROCK2 inhibitor |
|  | Scleroderma renal crisis within 4 months prior to enrolment |
|  | FVC ≤ 50% Predicted |

ACR, American College of Rheumatology and European League Against Rheumatism; EULAR, American College of Rheumatology and European League Against Rheumatism; FVC, forced vital capacity; mRSS, modified Rodnan skin score; QTc, QT interval; ROCK, Rho-associated coiled coil-containing protein kinase; ULN, upper limit of normal.

**Supplementary Table S2:** Objectives and endpoints of the study

| **Objectives** | **Endpoints** |
| --- | --- |
| **Primary** | |
| To evaluate the efficacy of belumosudil compared to placebo using the CRISS at Week 24 | - Number of participants with CRISS score 0.60 at Week 24 - Continuous CRISS score at Week 24 |
| **Secondary** | |
| - To assess the CRISS for each group at Week 52 - To evaluate the efficacy of belumosudil compared to placebo at Week 24 for:   mRSS, FVC, Physician Global Assessment, Patient Global Assessment, and SHAQ-DI | - Continuous CRISS score at Week 52 - Mean value, change from baseline and percentage improvement at Week 24 in mRSS - Mean value and change from baseline at Week 24 in FVC - Mean value, change from baseline, and percentage improvement at Week 24 in Physician Global Assessment - Mean value, change from baseline, and percentage improvement at Week 24 in Patient Global Assessment - Mean value, change from baseline, and percentage improvement at Week 24 in SHAQ-DI - Mean value and change from baseline at Week 24 in participants with ILD at Screening |
| To evaluate the efficacy of belumosudil at Week 52 compared to baseline for participants randomized to belumosudil for the parameters in the row above | - Mean value, change from baseline, and percentage improvement at Week 52 in mRSS - Mean value and change from baseline at Week 52 in FVC - Mean value, change from baseline, and percentage improvement at Week 52 in Physician Global Assessment - Mean value, change from baseline, and percentage improvement at Week 52 in Patient Global Assessment - Mean value, change from baseline, and percentage improvement at Week 52 in SHAQ-DI - Mean value and change from baseline at Week 52 in PFTs in participants with ILD at Screening |
| To assess changes in lung fibrosis, via HRCT, performed at baseline, Week 24, and Week 52 only in participants with ILD at Screening | Lung fibrosis assessed via HRCT at Week 24 and at Week 52 in participants with ILD at Screening |
| To assess the safety of belumosudil compared to placebo in participants with dcSSc by examining the percentage of participants with TEAEs (CTCAE version 5.0) | The safety endpoints included percentage of participants who experienced TEAEs and treatment-emergent serious adverse events (CTCAE version 5.0) |
| **Exploratory** | |
| To evaluate changes in biomarkers of endothelial cell dysfunction, fibrosis, immune system function, and cytokine alterations from participants receiving belumosudil or placebo | Changes in biomarkers of endothelial cell dysfunction, fibrosis, immune system function, and cytokine alterations from participants receiving belumosudil or placebo |
| To assess histology and gene expression from skin biopsies taken from participants at baseline, Week 24, and Week 52 | Histology and gene expression from skin biopsies taken from participants at baseline, Week 24, and Week 52 |

CRISS, Combined Response Index in diffuse cutaneous systemic sclerosis; CTCAE, common terminology criteria for adverse events; FVC, forced vital capacity; HRCT, high-resolution computerized tomography; ILD, interstitial lung disease; mRSS, modified Rodnan skin score; PFT, Pulmonary Function Tests; SHAQ-DI, scleroderma health assessment questionnaire disability-index; TEAE, treatment-emergent adverse events.

**Supplementary Table S3:** Change from baseline at Week 24 evaluated using the MMRM model on CRISS components in the mITT population

|  | **Belumosudil 200 mg QD** | **Belumosudil 200 mg BID** | **Placebo** |
| --- | --- | --- | --- |
|  | **(n=11)** | **(n=12)** | **(n=11)** |
| **mRSS** |  |  |  |
| LSM (95% CI) | -9.2 (-12.2, -6.2) | -4.0 (-7.0, -1.0) | -8.6 (-11.4, -5.8) |
| LSM Difference (Diff ; 95% CI) versus placebo | -0.6 (-4.7, 3.6) | 4.6 (0.5, 8.8) | - |
| p-value vs placebo | 0.7710 | 0.0308 | - |
| **FVC** |  |  | - |
| LSM (95% CI) | 0.6 (-4.0, 5.2) | -2.3 (-7.2, 2.5) | -0.8 (-5.2, 3.6) |
| LSM Difference (Diff ; 95% CI) versus placebo | 1.4 (-4.9, 7.7) | -1.5 (-8.1, 5.0) | - |
| p-value vs placebo | 0.6533 | 0.6338 | - |
| **Physician Global Assessment** |  |  |  |
| LSM (95% CI) | 22.4 (10.0, 34.8) | -0.8 (-13.2, 11.6) | 7.8 (-4.1, 19.6) |
| LSM Difference (Diff ; 95% CI) versus placebo | 14.6 (-2.5, 31.8) | -8.6 (-25.7, 8.6) | - |
| p-value vs placebo | 0.0916 | 0.3146 | - |
| **Patient Global Assessment** |  |  |  |
| LSM (95% CI) | -5.8 (-22.4, 10.8) | 3.5 (-13.1, 20.1) | 4.2 (-11.4, 19.8) |
| LSM Difference (Diff ; 95% CI) versus placebo | -10.0 (-32.8, 12.8) | -0.7 (-23.5, 22.1) | - |
| p-value vs placebo | 0.3753 | 0.9481 | - |
| **SHAQ-DI** |  |  |  |
| LSM (95% CI) | -0.19 (-0.45, 0.07) | -0.1 (-0.37, 0.15) | -0.15 (-0.39, 0.09) |
| LSM Difference (Diff ; 95% CI) versus placebo | -0.036 (-0.392, 0.320) | 0.039 (-0.317, 0.395) | - |
| p-value vs placebo | 0.8387 | 0.8234 | - |

BID, twice daily; CI, confidence interval; FVC, forced vital capacity; LSM, least square mean; MMRM, mixed-model repeated measures; mRSS, modified Rodnan skin score; n/N, number of patients; QD, once daily; SHAQ-DI, scleroderma health assessment questionnaire disability-index.

**Supplementary Table S4:** Summary of pulmonary function test results of patients with interstitial lung disease from baseline at Week 24

| **Median (range)** | **Double-blind period*** | | | **Open-label period**^#^ | |
| --- | --- | --- | --- | --- | --- |
|  | **Belumosudil 200 mg QD** | **Belumosudil 200 mg BID** | **Placebo** | **Belumosudil  200 mg QD** | **Belumosudil 200 mg BID** |
| Forced vital capacity, (%) | n=4 | n=5 | n=5 | n=6 | n=6 |
|  | -4.5 (-19 – 19) | -3.0 (-10 – 5) | 1.0 (-3 – 3) | -5.5 (-12 – -2) | 2.0 (-5 – 18) |
| Total lung capacity, (%) | n=3 | n=5 | n=4 | n=5 | n=5 |
|  | -14.0 (-14 – -6) | -2.0 (-8 – 2) | 11.0 (3 – 17) | -11.0 (-25 – -3) | 0.0 (-14 – 6) |
| Diffuse capacity of lung for CO (DLco), (%) | n=3 | n=5 | n=3 | n=6 | n=6 |
|  | -2.0 (-18 – 1) | 5.0 (-3 – 14) | 0.0 (-2 – 6) | -7.0 (-18 – 7) | 2.0 (-7 – 15) |

* For double-blinded period, Belumosudil 200 mg QD or Belumosudil 200 mg BID or placebo were administered for the first 28 weeks.

^#^ For open-label period, Belumosudil 200 mg QD or Belumosudil 200 mg BID were administered from Week 28 to 52.

BID, twice daily; n, number of patients with interstitial lung disease; QD, once daily

**Supplementary Table S5:** Summary of adverse events (AEs) reported in the safety population

| **N (%)** | **Double-blinded period*** | | | **Open-label period**^#^ | |
| --- | --- | --- | --- | --- | --- |
|  | **Belumosudil 200 mg QD** | **Belumosudil 200 mg BID** | **Placebo** | **Belumosudil 200 mg QD** | **Belumosudil 200 mg BID** |
|  | **(n=11)** | **(n=12)** | **(n=12)** | **(n=15)** | **(n=16)** |
| Patients with AEs | 11 (100.0) | 11 (91.7) | 11 (9.7) | 14 (93.3) | 14 (87.5) |
| Patients with TEAEs^ | 11 (100.0) | 11 (91.7) | 11 (9.7) | 14 (93.3) | 14 (87.5) |
| Patients with serious TEAEs | 2 (18.2) | 0 | 3 (25.0) | 0 | 1 (6.3) |
| Patients with Grade ≥3 TEAEs | 0 | 1 (8.3) | 2 (16.7) | 1 (6.7) | 2 (12.5) |
| Patients with TRAEs | 7 (63.6) | 6 (50.0) | 5 (41.7) | 0 | 5 (31.3) |

* For double-blinded period, belumosudil 200 mg QD or belumosudil 200 mg BID or placebo were administered for the first 28 weeks.

# For open-label period, belumosudil 200 mg QD or belumosudil 200 mg BID or placebo were administered from Week 28 to 52.

^TEAEs: any AE occurring or worsening in severity after the first administration of study medication

BID, twice daily; QD, once daily; n, number of patients; TEAE, treatment-emergent AE; TRAE, treatment-related AE

**Supplementary Table S6:** Summary of Grade ≥3 treatment-emergent adverse events (TEAEs)* reported in the safety population

| **N (%)** | **Double-blinded period^#^** | | | **Open-label period^** | |
| --- | --- | --- | --- | --- | --- |
|  | **Belumosudil  200 mg QD** | **Belumosudil 200 mg BID** | **Placebo** | **Belumosudil  200 mg QD** | **Belumosudil  200 mg QD** |
|  | **(n=11)** | **(n=12)** | **(n=12)** | **(n=15)** | **(n=16)** |
| **Patients with at least one Grade ≥3 TEAE** | 0 | 1 (8.3) | 2 (16.7) | 1 (6.7) | 2 (12.5) |
| **Cardiac disorders** | 0 | 0 | 0 | 0 | 2 (12.5) |
| Bradycardia | 0 | 0 | 0 | 0 | 1 (6.3) |
| Cardiac failure congestive | 0 | 0 | 0 | 0 | 1 (6.3) |
| Ischaemic cardiomyopathy | 0 | 0 | 0 | 0 | 1 (6.3) |
| **Infections and infestations** | 0 | 1 (8.3) | 0 | 0 | 0 |
| COVID-19 | 0 | 1 (8.3) | 0 | 0 | 0 |
| **Respiratory, thoracic, and mediastinal disorders** | 0 | 0 | 1 (8.3) | 0 | 1 (6.3) |
| Pulmonary oedema | 0 | 0 | 1 (8.3) | 0 | 0 |
| Dyspnoea exertional | 0 | 0 | 0 | 0 | 1 (6.3) |
| **Vascular disorders** | 0 | 0 | 0 | 1 (6.7) | 0 |
| Hypertension | 0 | 0 | 0 | 1 (6.7) | 0 |
| **Blood and lymphatic system disorders** | 0 | 0 | 1 (8.3) | 0 | 0 |
| Anaemia | 0 | 0 | 1 (8.3) | 0 | 0 |
| **Renal and urinary disorders** | 0 | 0 | 1 (8.3) | 0 | 0 |
| Scleroderma renal crisis | 0 | 0 | 1 (8.3) | 0 | 0 |

*TEAEs: any AE occurring or worsening in severity after the first administration of study medication

^#^ For double-blinded period, belumosudil 200 mg QD or belumosudil 200 mg BID or placebo were administered for the first 28 weeks.

^For open-label period, belumosudil 200 mg QD or belumosudil 200 mg BID or placebo were administered from Week 28 to 52.

BID, twice daily; COVID, coronavirus disease; N/n, number of patients; QD, once daily.
