# Supplementary material for: Belumosudil in diffuse cutaneous systemic sclerosis: a randomized, double-blind, open-label extension, placebo-controlled, phase 2 study
Source: Rheumatology (Oxford). 2025 Mar 14;64(7):4299–308. doi: 10.1093/rheumatology/keaf062 (PMC12212915; doi:10.1093/rheumatology/keaf062)
Supplement: keaf062_Supplementary_Data [file keaf062_supplementary_data.zip › keaf062_Supplementary_Data/rhe-24-0882-File003.docx]

**
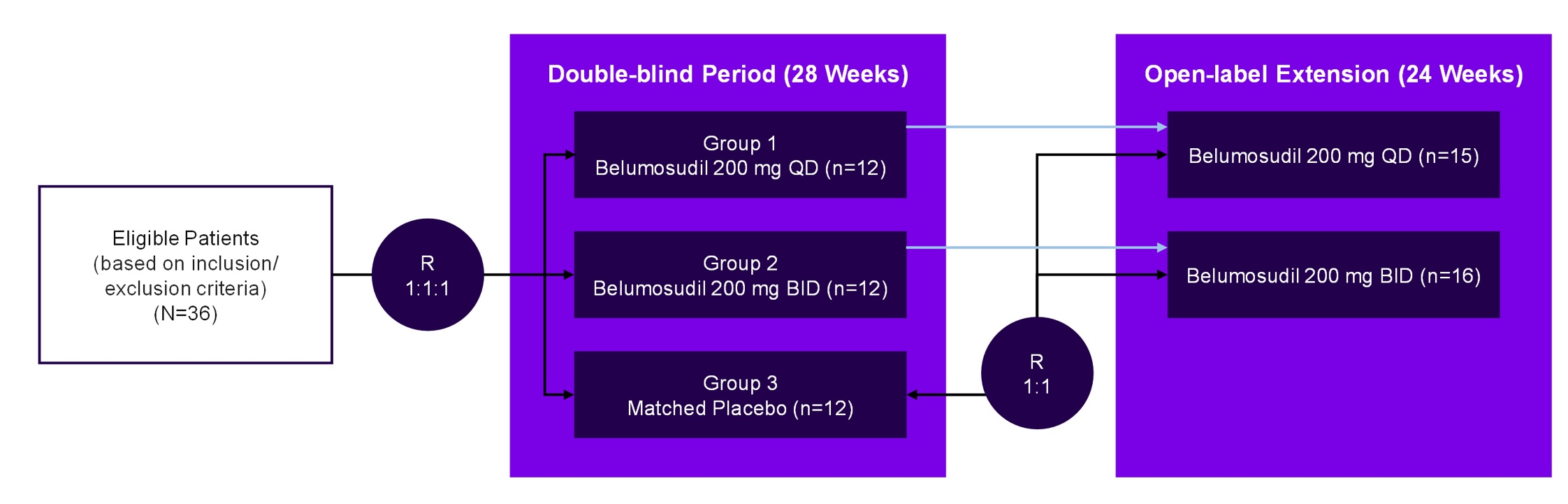
**

**Supplementary Figure S1:** Study design schema.

BID, twice daily; dcSSc, diffuse cutaneous systemic sclerosis; n, number of patients; QD, once daily; R, randomization.

**
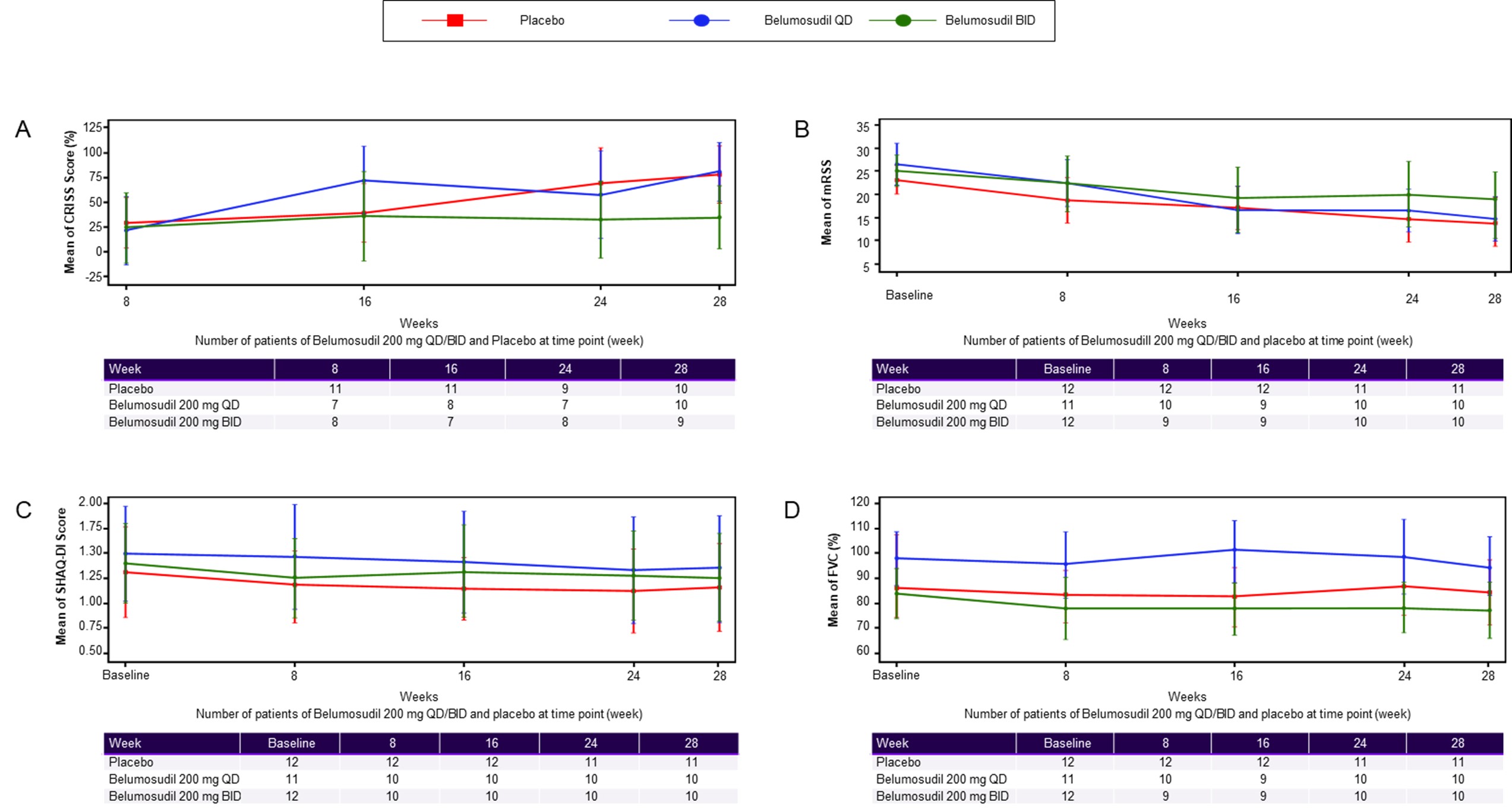
**

**Supplementary Figure S2:** Analysis of CRISS*, FVC, mRSS and SHAQ-DI in the double-blinded period up to Week 28 (mITT population). A) Mean CRISS score; B) Mean mRSS; C) Mean SHAQ-DI; and D) Mean FVC (%)

*CRISS Score >0.20 was the minimally detectable difference; CRISS score >0.60 was the minimally important difference. Only included scheduled visits up to Week 28.

BID, twice daily; CRISS, Combined Response Index in Diffuse Cutaneous Systemic Sclerosis; FVC, forced vital capacity; mITT, modified intent-to-treat; PBO, placebo for the first 28 weeks. mRSS, modified Rodnan skin score; QD, once daily; SHAQ-DI, scleroderma health assessment questionnaire disability-index


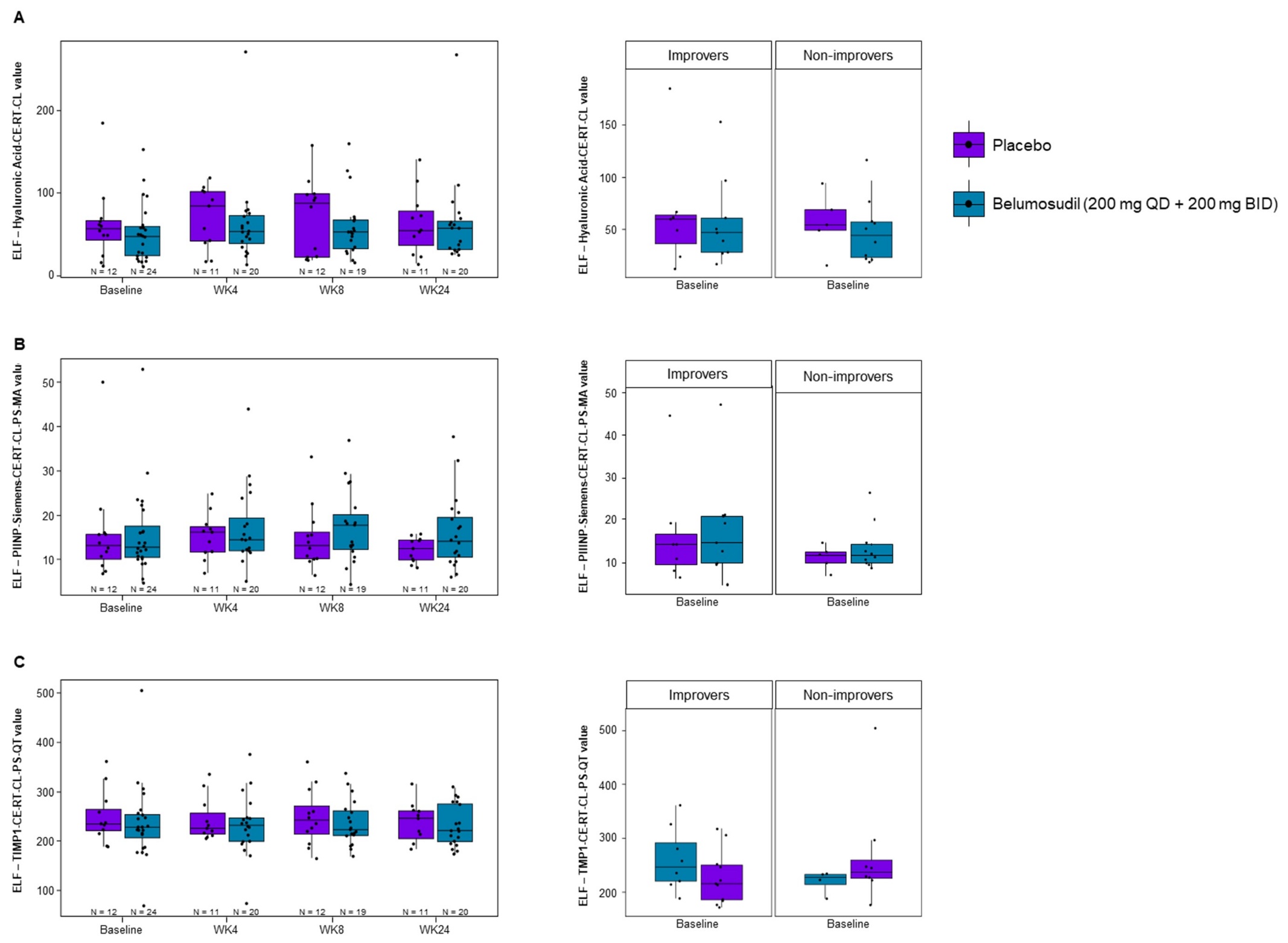


**Supplementary Figure S3:** Comparative analysis of peripheral fibrosis biomarker using the ELF score in the belumosudil 200 mg (pooled QD + BID)-treated group and the placebo group. A) ELF_HA score. B) ELF_PIIINP score. C) ELF_TIMP-1 score. Box center and upper/lower lines indicate the median and upper/lower quartile, respectively. Vertical lines above and below the box indicate 1.5 times the interquartile range.

BID, twice daily; ELF, enhanced liver fibrosis; HA, hyaluronic acid; N, number of patients; PIIINP, type III procollagen peptide; QD, once daily; SD, standard deviation; TIMP-1; tissue inhibitor of matrix metalloproteinase 1; WK, week
